# Variation of all-cause and cause-specific mortality with body mass index in one million Swedish parent-son pairs: An instrumental variable analysis

**DOI:** 10.1371/journal.pmed.1002868

**Published:** 2019-08-09

**Authors:** Kaitlin H. Wade, David Carslake, Per Tynelius, George Davey Smith, Richard M. Martin

**Affiliations:** 1 Population Health Sciences, Bristol Medical School, Faculty of Health Sciences, University of Bristol, Bristol, United Kingdom; 2 Medical Research Council Integrative Epidemiology Unit at the University of Bristol, Bristol, United Kingdom; 3 Department of Public Health Sciences, Karolinska Institutet, Stockholm, Sweden; 4 National Institute for Health Research Bristol Biomedical Research Centre, Bristol, United Kingdom; Harvard Medical School, UNITED STATES

## Abstract

**Background:**

High body mass index (BMI) is associated with mortality, but the pervasive problem of confounding and reverse causality in observational studies limits inference about the direction and magnitude of causal effects. We aimed to obtain estimates of the causal association of BMI with all-cause and cause-specific mortality.

**Methods and findings:**

In a record-linked, intergenerational prospective study from the general population of Sweden, we used two-sample instrumental variable (IV) analysis with data from 996,898 fathers (282,407 deaths) and 1,013,083 mothers (153,043 deaths) and their sons followed up from January 1, 1961, until December 31, 2004. Sons’ BMI was used as the instrument for parents’ BMI to compute hazard ratios (HRs) for risk of mortality per standard deviation (SD) higher parents’ BMI. Using offspring exposure as an instrument for parents’ exposure is unlikely to be affected by reverse causality (an important source of bias in this context) and reduces confounding. IV analyses supported causal associations between higher BMI and greater risk of all-cause mortality (HR [95% confidence interval (CI)] per SD higher fathers’ BMI: 1.29 [1.26–1.31] and mothers’ BMI: 1.39 [1.35–1.42]) and overall cancer mortality (HR per SD higher fathers’ BMI: 1.20 [1.16–1.24] and mothers’ BMI: 1.29 [1.24–1.34]), including 9 site-specific cancers in men (bladder, colorectum, gallbladder, kidney, liver, lung, lymphatic system, pancreas, and stomach) and 11 site-specific cancers in women (gallbladder, kidney, liver, lung, lymphatic system, ovaries, pancreas, stomach, uterus, cervix, and endometrium). There was evidence supporting causal associations between higher BMI in mothers and greater risk of mortality from kidney disease (HR: 2.17 [1.68–2.81]) and lower risk of mortality from suicide (HR: 0.77 [0.65–0.90]). In both sexes, there was evidence supporting causal associations between higher BMI and mortality from cardiovascular diseases (CVDs), stroke, diabetes, and respiratory diseases. We were unable to test the association between sons’ and mothers’ BMIs (as mothers’ data were unavailable) or whether the instrument was independent of unmeasured or residual confounding; however, the associations between parents’ mortality and sons’ BMI were negligibly influenced by adjustment for available confounders.

**Conclusions:**

Consistent with previous large-scale meta-analyses and reviews, results supported the causal role of higher BMI in increasing the risk of several common causes of death, including cancers with increasing global incidence. We also found positive effects of BMI on mortality from respiratory disease, prostate cancer, and lung cancer, which has been inconsistently reported in the literature, suggesting that the causal role of higher BMI in mortality from these diseases may be underestimated. Furthermore, we expect different patterns of bias in the current observational and IV analyses; therefore, the similarities between our findings from both methods increases confidence in the results. These findings support efforts to understand the mechanisms underpinning these effects to inform targeted interventions and develop population-based strategies to reduce rising obesity levels for disease prevention.

## Abstract 2

Kaitlin Wade and colleagues investigate associations between BMI and cause-specific mortality in a large cohort of parent-son pairs.

## Author summary

**Why was this study done?:**

- Whilst high body mass index (BMI) clearly increases the risk of mortality, there have been inconsistencies in the literature, which mainly rely on observational epidemiological studies that are limited in their ability to draw causal inferences.
- To overcome the pervasive problem of confounding and reverse causation in such studies, we aimed to obtain more reliable causal estimates of the association of BMI and both all-cause and cause-specific mortality using two-sample instrumental variable (IV) analyses.

**What did the researchers do and find?:**

- In a large, record-linked, intergenerational prospective study comprising data from the general population of Sweden from 996,898 fathers (282,407 deaths) and 1,013,082 mothers (153,043 deaths) and their sons, we used sons’ BMI as an instrument for parents’ BMI to compute hazard ratios (HRs) for the risk of mortality per standard deviation (SD) higher parents’ BMI.
- IV analyses supported the causal role of higher BMI and greater risk of all-cause mortality and mortality from cardiovascular diseases (CVDs), stroke, diabetes, respiratory diseases, and cancer, the latter including 9 site-specific cancers in men (bladder, colorectum, gallbladder, kidney, liver, lung, lymphatic system, pancreas, and stomach) and 11 site-specific cancers in women (gallbladder, kidney, liver, lung, lymphatic system, ovaries, pancreas, stomach, uterus, cervix, and endometrium).
- There was also evidence supporting a causal association between higher BMI in mothers and greater risk of mortality from kidney disease and lower risk of mortality from suicide.

**What do these findings mean?:**

- For cancer-specific mortality, many IV-derived effect estimates were in the same direction and of greater magnitude as those derived from previous large-scale meta-analyses and reviews focusing on both mortality outcomes and the development of specific cancers.
- Results also implied a positive effect of BMI on mortality from respiratory disease, prostate cancer, and lung cancer, which has been inconsistently reported in the literature, suggesting that the causal role of higher BMI in mortality from these diseases may be underestimated.
- These results support efforts to reduce obesity levels for disease prevention, particularly cancer.

## Introduction

The prevalence of obesity is rising [1–3], and severe obesity (body mass index [BMI] ≥35 kg/m^2^) is associated with a greater risk of death [4–7]. However, the magnitude of this relationship over the range of BMI, whether or not higher BMI is a causal determinant of both risk and progression of several cancers [8–10] or could protect against some diseases (i.e., cancers of the lung [11,12] and prostate [8,13] and respiratory diseases [4]) is debated [5,14,15]. A systematic review concluded that, relative to normal weight (18.5–24.9 kg/m^2^), the risk of all-cause mortality was greater with severe obesity, but overweight (25.0–29.9 kg/m^2^) was protective and moderate obesity (30–34.5 kg/m^2^) did not alter mortality risk (suggesting a ‘J-shaped’ association) [14]. If mortality is elevated at only very high BMI, rather than in a linear fashion, this would undermine public health efforts aimed at shifting the entire BMI distribution downwards to reduce the adverse consequences of obesity [16].

There are at least 2 important alternative explanations of this observed J-shaped association: (i) confounding by lifestyle and/or behavioural factors, such as smoking (i.e., smokers tend to be leaner and have higher mortality than nonsmokers); and (ii) reverse causality (i.e., people losing weight due to existing [and potentially undiagnosed and/or undetected] illness) [17–19]. These effects could generate spurious positive associations of normal (or under-) weight with mortality or mask effects of moderate obesity. Excluding or adjusting for confounding factors and removal of deaths occurring in the first years of follow-up are attempts to overcome such limitations [20]. Indeed, excluding ever-smokers and those who died during the first 4 years of follow-up generated a linear positive relationship between BMI and all-cause mortality risk, with the lowest mortality at BMI <19 kg/m^2^ [15]. In another study of 1.46 million never-smokers with >10-years follow-up (not included in the aforementioned review [14]), the lowest mortality risk was observed in the recommended normal BMI range [5]. Despite this, measurement error and unmeasured factors can still lead to residual confounding [21] and biased estimations [22,23]. To compound the confusing observational literature, a recent United States–based study observed lower all-cause mortality risk in people who were overweight, but not obese, versus normal weight, even in never-smokers or those with stable weight during follow-up (i.e., excluding those with weight loss caused by illness) [24].

Instrumental variable (IV) analysis aims to overcomes biases inherent in observational epidemiology and hence improve assessment of causality [25,26]. One application of IV analyses is the use of offspring exposures as ‘instruments’ for parents’ exposures, for which we and others have provided justification [20,23,27–29]. Whilst offspring exposures may not be independent of confounding factors, they are robustly related to parents’ own exposures and only affect the outcome (here, parental mortality) via the parental exposure of which they are acting as proxies (i.e., likely protected against reverse causality). We conducted a record-linked, intergenerational prospective cohort study based on a large-scale Swedish cohort. We analysed data using IV analysis, in which sons’ BMI was used as a proxy for parents’ BMI to provide unbiased causal estimates of the association between parents’ BMI and all-cause and cause-specific mortality.

## Methods

### Study population and data linkage

The Swedish Multi-Generation Register was used to identify all boys born in Sweden in 1951 through 1980 and their biological parents, using the unique identity numbers (IDs) given to all citizens and individuals with permanent permission to live in Sweden [20,28]. Sons’ IDs were used to obtain their height and weight, which were measured at conscription examinations between September 1969 and December 2001, at a mean age of 18 years (range 16–25). Fathers also underwent conscription examinations for height and weight between September 1, 1969 and May 1, 1991. Father–son pairs were defined using these conscription records and Multi-Generation Register, which included the unique national IDs for each son and his biological parents. The parents’ IDs were matched to the Swedish Cause of Death Register, providing dates and causes of parents’ deaths between January 1, 1961 and December 31, 2004. The underlying cause of death was recorded by the international classification of diseases (ICD; versions 7–10) codes on death certificates (S1 Table). The study was approved by the Research Ethics Committee of the University of Bristol Faculty of Health Sciences. For details on study population, data linkage, and measurements, see S1 Text.

### Statistical analysis

Our a priori analysis plan was to repeat our previously published study using extended follow-up and the more powerful IV methods applied in our subsequent studies [20,30] (see S1 Text). The distributions of fathers’ and sons’ BMI, height, and smoking, and parents’ ages, education, and occupational socioeconomic index (SEI) were examined in groups defined by quintiles of the sons’ or fathers’ BMI through logistic or linear regression, as appropriate. These analyses used all sons available in the analysis of either parent.

#### Conventional Cox proportional hazards regression

Within a subset of father–son pairs in which fathers also had data on BMI, conscription office and dates of birth, and examination, Cox proportional hazards regression models were used to estimate conventional hazard ratios (HRs) for all-cause and cause-specific mortality per standard deviation (SD; 2.90 kg/m^2^) of fathers’ BMI. Each father’s age was the time axis; models were thus adjusted for fathers’ ages. These and all subsequent models were conducted with and without adjustment for parents’ educational and occupational SEI and were restricted to those conditions responsible for >40 deaths.

#### IV analysis

Within the same subset of data, we performed a conventional one-sample IV analysis using the ratio method by estimating (i) the HR of all-cause and cause-specific deaths per SD of sons’ BMI (2.90 kg/m^2^) using Cox proportional hazards regression (numerator) and (ii) the association between fathers’ and sons’ BMI (denominator). The causal HR per SD of fathers’ BMI was derived by exponentiating the ratio between the natural logarithm of the HR from (i) and the mean difference from (ii), using the same adjustments. Confidence intervals (CIs) were calculated using Taylor series expansions. Instrument strength was assessed using the F-statistic of the denominator. The HRs per SD of fathers’ BMI from the conventional Cox regression were compared with the corresponding HRs from the IV ratio method by applying a Durbin–Wu–Hausman test to the log(HR) and their standard errors. We also generated alternative IV estimates using Poisson regression within strata of fathers’ ages and Stata’s qvf command (S1 Text).

We generated two-sample IV estimates of the HR per SD of fathers’ BMI using the same ratio method and sons’ BMI as the instrument using all available data for the numerator (i.e., not within the subset of data in which all information on sons’ and fathers’ BMI and mortality was available) [25,26].

Finally, with the additional assumption that the mother–son BMI association was equivalent to the father–son BMI association, we used the same two-sample methodology (as described above) to estimate the HR per SD of mothers’ BMI (using sons’ BMI as an instrument), even though mothers’ BMI was not measured. After restricting the data to those sons used in the analysis of both their parents’ mortality, we used 1,000 bootstrap resamples to compare the HR for mothers’ and fathers’ mortality per SD of sons’ BMI.

For each mortality outcome, we present HRs per SD of own BMI (i.e., of either fathers or mothers) from conventional Cox regression and IV analyses using both one- and two-sample methodology. Whilst one-sample IV analyses allowed the direct comparison with conventional Cox regression, the two-sample IV analyses used all available data to increase the precision of causal estimates and can therefore be interpreted as our best causal estimate of the relationship between BMI and mortality. For completeness and transparency, we also present HRs per SD of sons’ BMI (i.e., the numerator of the ratio IV estimate). All statistical analyses were performed using Stata 14.2 on a desktop machine and Stata 12.1 on the University of Bristol’s Blue Crystal high power computing cluster.

## Results

**Fig 1:**
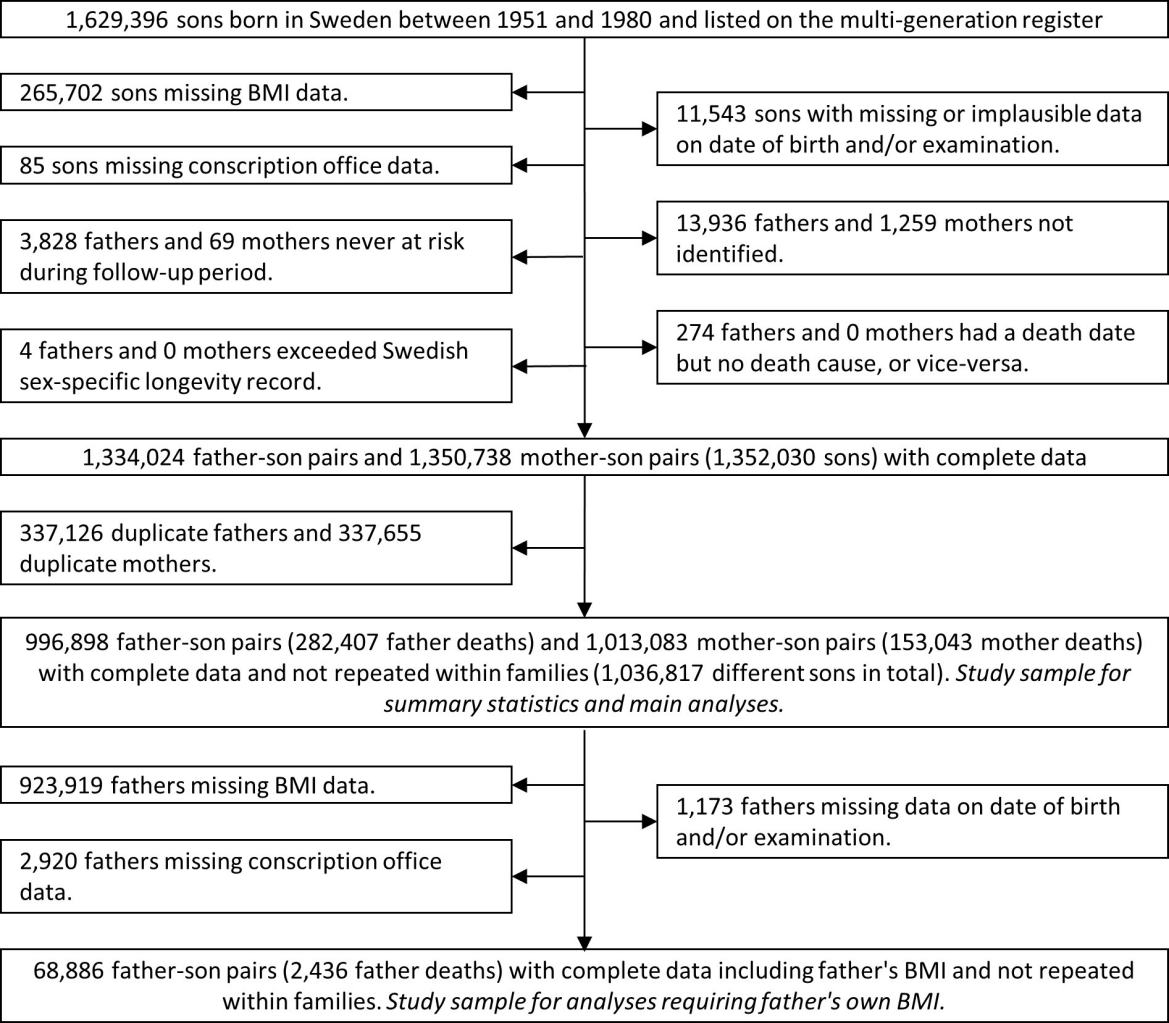
Path of participants through the study. BMI, body mass index.

Of the 1,629,396 boys identified from the Swedish Multi-Generation Register, the available sample for current analyses included 996,898 father–son pairs (282,407 deaths) and 1,013,083 mother–son pairs (153,043 deaths) for 1,036,817 different sons and including 973,164 complete trios (Fig 1). Fathers’ BMI was associated with sons’ BMI (regression coefficient: 0.62 kg/m^2^ per SD of sons’ BMI; 95% CI: 0.60–0.64). Sons with higher BMI had parents who were less likely to spend >10 years in full-time education or be in nonmanual employment (Table 1). Sons with higher BMI were less likely to smoke but had fathers who were more likely to smoke. Anthropometric variables and smoking were more strongly associated with fathers’ BMI than they were with sons’ BMI, but measured potential socioeconomic confounders were similarly associated with fathers’ and sons’ BMI (S2 Table and S3 Table).

**Table 1:** Characteristics of the sons and parents according to quintiles of sons’ BMI.

Subject	Variable	Quintile of sons’ BMI					Mean difference or odds ratio (95% CI)	*N*
		1st	2nd	3rd	4th	5th		
**Sons**	BMI^a^^,^^b^ (kg/m^2^)	18.5	20.2	21.3	22.7	26.2	2.90 (2.90–2.90)	1,036,817
	Height^a^^,^^b^ (cm)	179.6	179.4	179.3	179.1	179.0	–0.19 (−0.20 to −0.18)	1,036,817
	Smokers^b^^,^^c^ (%)	64%	61%	59%	57%	56%	0.91 (0.89–0.93)	29,508
	BMI^a^^,^^b^ (kg/m^2^)	20.3	20.9	21.2	21.6	22.2	0.62 (0.60–0.64)	73,982
	Height^a^^,^^b^ (cm)	178.4	178.4	178.2	178.2	178.2	–0.09 (−0.13 to −0.05)	74,001
**Fathers**	Date of birth^a^	1936.4	1935.3	1934.9	1934.9	1935.8	0.003 (−0.02 to 0.03)	1,025,639
	Smokers^b^^,^^c^ (%)	62%	64%	62%	64%	67%	1.09 (1.06–1.12)	17,254
	Age at sons’ birth (years)^a^	30.5	30.5	30.5	30.5	30.5	–0.003 (−0.02 to 0.01)	1,025,639
	Educated >10 years^c^ (%)	57%	56%	55%	53%	48%	0.88 (0.88–0.88)	973,704
	In nonmanual work^c^ (%)	51%	51%	50%	49%	42%	0.86 (0.86–0.87)	858,447
**Mothers**	Date of birth	1939.4	1938.4	1938.0	1938.0	1939.0	0.05 (0.03–0.07)	1,035,861
	Age at sons’ birth (years)^a^	27.4	27.4	27.4	27.4	27.3	–0.05 (−0.06 to −0.04)	1,035,861
	Educated >10 years^c^ (%)	56%	55%	55%	53%	50%	0.92 (0.91–0.92)	1,016,365
	In nonmanual work^c^ (%)	50%	50%	49%	47%	42%	0.88 (0.88−0.88)	753,046

^a^Continuous variables are summarised as means in each quintile and linear regression produced mean differences per SD (2.90 kg/m^2^) of BMI preadjusted for each son’s age at examination, conscription office, and secular trends (date of birth).

^b^Measured at preconscription medical examination. Smoking was only recorded at examinations in 1969 through 1970.

^c^Binary variables are summarised as percentages in each quintile and logistic regression produced odds ratios per SD (2.90 kg/m^2^) of BMI preadjusted for each son’s age at examination, conscription office, and secular trends (date of birth).

**Abbreviations:** BMI, body mass index; CI, confidence interval; SD, standard deviation

Within a subset of father–son pairs in which fathers also had data on BMI, conscription office and dates of birth, and examination, adjusted Cox regression showed that fathers with a higher BMI had higher mortality risk from all-causes, cardiovascular disease (CVD), coronary heart disease (CHD), stroke, overall cancer, and cancers of the brain and colorectum. There was also evidence that fathers with a higher BMI had lower mortality risk from external causes and, specifically, suicide (Table 2). Sons’ BMI was associated with fathers’ BMI (adjusted regression coefficient: 0.21 kg/m^2^ per SD of fathers’ BMI; 95% CI: 0.20–0.22; F-statistic = 673.8), suggesting that sons’ BMI was a strong instrument. Using one-sample IV analyses, the point estimates for associations of fathers’ BMI with all mortality outcomes were mostly stronger compared with conventional Cox regression (Table 2). The precision of the effect estimates was low for some IV analyses, and the Durbin–Wu–Hausman tests did not find strong evidence for a difference. IV estimates using stratified Poisson regression were almost identical to main analyses (S4 Table).

**Table 2:** A comparison of models for estimating associations of fathers’ mortality per SD (2.90 kg/m^2^) of BMI at conscription (sons’ or own) in the subset of fathers with BMI data (*N* = 68,886).

Cause of death	Deaths	HR^a^ (95% CI) per SD of own BMI, unadjusted	HR^b^ (95% CI) per SD of own BMI, adjusted	HR^c^ (95% CI) per SD of sons’ BMI, adjusted	IV HR^d^ (95% CI) per SD of own BMI, adjusted	*p*-Value^e^ for comparison of own BMI with IV HR
**All cause**	2,436	1.08 (1.03–1.13)	1.07 (1.03–1.12)	1.02 (0.99–1.06)	1.10 (0.94–1.29)	0.746
**CVD**	436	1.36 (1.23–1.49)	1.33 (1.21–1.47)	1.09 (1.01–1.18)	1.52 (1.06–2.19)	0.455
**CHD**	243	1.43 (1.27–1.62)	1.40 (1.24–1.59)	1.14 (1.04–1.26)	1.90 (1.19–3.02)	0.189
**Stroke**	89	1.27 (1.02–1.58)	1.25 (1.01–1.55)	1.09 (0.92–1.29)	1.52 (0.69–3.38)	0.614
**Respiratory diseases**	42	1.14 (0.81–1.60)	1.11 (0.80–1.55)	0.91 (0.69–1.20)	0.63 (0.17–2.42)	0.397
**External causes**	1,109	0.88 (0.82–0.95)	0.89 (0.83–0.95)	0.98 (0.93–1.03)	0.92 (0.72–1.18)	0.752
**Suicide**	495	0.84 (0.75–0.94)	0.85 (0.76–0.95)	0.93 (0.85–1.00)	0.69 (0.47–1.02)	0.272
**Cancer**	444	1.22 (1.10–1.35)	1.22 (1.10–1.34)	1.04 (0.96–1.13)	1.23 (0.84–1.79)	0.963
**Brain cancer**	66	1.29 (1.00–1.66)	1.32 (1.03–1.71)	0.94 (0.75–1.18)	0.75 (0.25–2.21)	0.287
**Colorectal cancer**	43	1.56 (1.19–2.06)	1.54 (1.17–2.02)	1.07 (0.83–1.37)	1.36 (0.42–4.42)	0.831
**Lung cancer**	63	1.01 (0.75–1.35)	1.00 (0.75–1.34)	1.00 (0.80–1.24)	0.98 (0.34–2.77)	0.962
**Lymphatic cancer**	67	1.25 (0.97–1.61)	1.24 (0.96–1.59)	1.02 (0.83–1.25)	1.09 (0.41–2.92)	0.799

^a^HR from Cox regression of fathers’ mortality per SD (2.90 kg/m^2^) of own BMI. Each father’s age was the time axis.

^b^HR from Cox regression of fathers’ mortality per SD (2.90 kg/m^2^) of own BMI, adjusted for educational and occupational SEI. Each father’s age was the time axis.

^c^HR from Cox regression of fathers’ mortality per SD (2.90 kg/m^2^) of sons’ BMI, adjusted for educational and occupational SEI. Each father’s age was the time axis. This can be interpreted as the numerator of the ratio estimate for IV analyses.

^d^HR from a ratio method IV estimate with sons’ BMI as the instrument for fathers’ BMI, adjusted for educational and occupational SEI.

^e^HR from the conventional analysis of fathers’ BMI were compared with those from the ratio method IV using a Durbin–Wu–Hausman test.

These analyses were all restricted entirely to those fathers with BMI data (*N* = 68,886) and to conditions causing at least 40 fathers’ deaths within this subset. BMI was preadjusted for secular trends, conscription office and age at examination.

**Abbreviations:** BMI, body mass index; CHD, coronary heart disease; CI, confidence interval; CVD, cardiovascular disease; HR, hazard ratio; IV, instrumental variable; SD, standard deviation; SEI, socioeconomic index

Using all available data (both for BMI and mortality outcomes in sons and parents), two-sample IV analyses supported the causal association of higher BMI on greater risk of all-cause mortality (HR [95% CI] per SD of fathers’ BMI: 1.29 [1.26–1.31]) and mortality from CVD (HR: 1.47 [1.43–1.51]), CHD (HR: 1.55 [1.50–1.61]), aortic aneurysm (HR: 1.41 [1.23–1.61]), stroke (HR: 1.22 [1.15–1.31]), diabetes (HR: 2.40 [2.10–2.74]), respiratory diseases (HR: 1.13 [1.04–1.22]), overall cancer (HR: 1.20 [1.16–1.24]), and cancers of the bladder (HR: 1.24 [1.02–1.51]), colorectum (HR: 1.27 [1.14–1.40]), gallbladder (HR: 1.32 [1.02–1.71]), kidney (HR: 1.38 [1.18–1.62]), liver (HR: 1.79 [1.47–2.17]), lung (HR: 1.13 [1.05–1.23]), lymphatic system (HR: 1.20 [1.08–1.34]), pancreas (HR: 1.45 [1.27–1.65]), and stomach (HR: 1.33 [1.17–1.53]) (Table 3).

**Table 3:** Cox proportional hazards models for fathers’ mortality with BMI (*N* = 996,898).

Cause of death	Deaths	HR^a^ (95% CI) per SD of sons’ BMI, unadjusted	HR^b^ (95% CI) per SD of sons’ BMI, adjusted	IV HR^c^ (95% CI) per SD of own BMI, adjusted
**All cause**	282,407	1.07 (1.07–1.08)	1.05 (1.05–1.06)	1.29 (1.26–1.31)
**CVD**	127,415	1.10 (1.10–1.11)	1.08 (1.08–1.09)	1.47 (1.43–1.51)
**CHD**	81,805	1.12 (1.11–1.13)	1.10 (1.09–1.10)	1.55 (1.50–1.61)
**Aortic aneurysm**	5,002	1.08 (1.05–1.11)	1.07 (1.04–1.11)	1.41 (1.23–1.61)
**Stroke**	22,022	1.06 (1.04–1.07)	1.04 (1.03–1.06)	1.22 (1.15–1.31)
**Diabetes**	4,278	1.22 (1.19–1.26)	1.20 (1.17–1.24)	2.40 (2.10–2.74)
**Kidney disease**	1,996	1.06 (1.01–1.11)	1.04 (1.00–1.09)	1.22 (0.98–1.51)
**Respiratory diseases**	14,015	1.05 (1.03–1.06)	1.03 (1.01–1.04)	1.13 (1.04–1.22)
**External causes**	25,651	1.02 (1.01–1.04)	1.00 (0.99–1.01)	0.99 (0.94–1.05)
**Suicide**	9,564	1.00 (0.98–1.02)	0.98 (0.96–1.00)	0.91 (0.83–1.00)
**Cancer**	79,393	1.05 (1.04–1.05)	1.04 (1.03–1.05)	1.20 (1.16–1.24)
**Bladder cancer**	2,475	1.05 (1.01–1.09)	1.05 (1.00–1.09)	1.24 (1.02–1.51)
**Brain cancer**	3,094	1.01 (0.98–1.05)	1.01 (0.97–1.05)	1.05 (0.89–1.25)
**Colorectal cancer**	8,929	1.06 (1.03–1.08)	1.05 (1.03–1.07)	1.27 (1.14–1.40)
**Gallbladder cancer**	1,362	1.07 (1.01–1.13)	1.06 (1.00–1.12)	1.32 (1.02–1.71)
**Kidney cancer**	3,494	1.08 (1.04–1.12)	1.07 (1.03–1.11)	1.38 (1.18–1.62)
**Liver cancer**	2,222	1.14 (1.09–1.18)	1.13 (1.08–1.18)	1.79 (1.47–2.17)
**Lung cancer**	14,636	1.04 (1.03–1.06)	1.03 (1.01–1.04)	1.13 (1.05–1.23)
**Lymphatic cancer**	8,207	1.05 (1.02–1.07)	1.04 (1.02–1.06)	1.20 (1.08–1.34)
**Malignant melanoma**	1,862	0.99 (0.95–1.04)	1.00 (0.95–1.04)	0.98 (0.78–1.23)
**Oesophageal cancer**	1,727	1.04 (0.99–1.09)	1.03 (0.98–1.08)	1.14 (0.90–1.44)
**Prostate cancer**	12,712	1.01 (0.99–1.03)	1.01 (0.99–1.03)	1.05 (0.96–1.15)
**Pancreatic cancer**	5,269	1.09 (1.06–1.12)	1.08 (1.05–1.11)	1.45 (1.27–1.65)
**Stomach cancer**	4,928	1.08 (1.05–1.11)	1.06 (1.03–1.09)	1.33 (1.17–1.53)
**Testicular cancer**	206	1.13 (1.00–1.28)	1.10 (0.97–1.25)	1.59 (0.88–2.88)
**Thyroid cancer**	223	1.01 (0.88–1.16)	1.00 (0.87–1.15)	0.98 (0.51–1.91)

^a^HR from Cox regression of fathers’ mortality per SD (2.90 kg/m^2^) of sons’ BMI (*N* = 996,898). BMI was preadjusted for secular trends, conscription office, and age at examination. Each father’s age was the time axis. This can be interpreted as the numerator of the ratio estimate for IV analyses.

^b^HR from Cox regression of fathers’ mortality per SD (2.90 kg/m^2^) of sons’ BMI (*N* = 996,898). BMI was preadjusted for secular trends, conscription office, age at examination, and both educational and occupational SEI. Each father’s age was the time axis. This can be interpreted as the numerator of the ratio estimate for IV analyses.

^c^HR from Cox regression of fathers’ mortality per SD (2.90kg/m^2^) of fathers’ BMI, using a two-sample IV approach with sons’ BMI as the instrument for fathers’ BMI, adjusted for educational and occupational SEI. The father–son association in BMI was estimated using the subset of data with fathers’ BMI (*N* = 68,886).

**Abbreviations:** CHD, coronary heart disease; CI, confidence interval; CVD, cardiovascular disease; HR, hazard ratio; IV, instrumental variable; SD, standard deviation; SEI, socioeconomic index

Assuming that mother–son and father–son BMI associations were identical, two-sample IV analyses provided evidence supporting the causal association between higher BMI and greater risk of maternal all-cause mortality (HR [95% CI] per SD of mothers’ BMI: 1.39 [1.35–1.42]) and mortality from CVD (HR: 1.62 [1.55–1.69]), CHD (HR: 1.85 [1.74–1.97]), aortic aneurysm (HR: 1.48 [1.16–1.89]), stroke (HR: 1.25 [1.15–1.35]), diabetes (HR: 4.07 [3.48–4.77]), kidney disease (HR: 2.17 [1.68–2.81]), respiratory disease (HR: 1.45 [1.30–1.62]), overall cancer (HR: 1.29 [1.24–1.34]), and cancers of the gallbladder (HR: 1.63 [1.35–1.96]), kidney (HR: 1.67 [1.36–2.06]), liver (HR: 1.52 [1.18–1.96]), lung (HR: 1.65 [1.48–1.83]), lymphatic system (HR: 1.27 [1.11–1.45]), ovaries (HR: 1.17 [1.02–1.34]), pancreas (HR: 1.39 [1.20–1.61]), stomach (HR: 1.30 [1.07–1.57]), uterus (HR: 1.52 [1.30–1.77]), cervix (HR: 1.29 [1.04–1.58]), and endometrium (HR: 1.89 [1.38–2.59]), and lower risk of external mortality causes (HR: 0.89 [0.81–0.98]), specifically suicide (HR: 0.77 [0.65–0.90]) (Table 4). Restricting to sons contributing to the analysis of both parents made no substantive difference (S5 Table).

**Table 4:** Cox proportional hazards models for mothers’ mortality with BMI (*N* = 1,013,083).

Cause of death	Deaths	HR^a^ (95% CI) per SD of sons’ BMI, unadjusted	HR^b^ (95% CI) per SD of sons’ BMI, adjusted	IV HR^c^ (95% CI) per SD of own BMI, adjusted
**All cause**	153,043	1.08 (1.07–1.08)	1.07 (1.07–1.08)	1.39 (1.35–1.42)
**CVD**	50,931	1.12 (1.11–1.13)	1.11 (1.10–1.12)	1.62 (1.55–1.69)
**CHD**	24,036	1.16 (1.14–1.17)	1.14 (1.12–1.15)	1.85 (1.74–1.97)
**Aortic aneurysm**	1,529	1.09 (1.04–1.15)	1.09 (1.03–1.14)	1.48 (1.16–1.89)
**Stroke**	14,498	1.06 (1.04–1.08)	1.05 (1.03–1.07)	1.25 (1.15–1.35)
**Diabetes**	2,600	1.37 (1.32–1.41)	1.34 (1.30–1.39)	4.07 (3.48–4.77)
**Kidney disease**	1,151	1.20 (1.13–1.26)	1.18 (1.11–1.24)	2.17 (1.68–2.81)
**Respiratory diseases**	7,842	1.09 (1.07–1.12)	1.08 (1.06–1.11)	1.45 (1.30–1.62)
**External causes**	9,449	0.98 (0.96–1.00)	0.98 (0.96–1.00)	0.89 (0.81–0.98)
**Suicide**	3,821	0.94 (0.91–0.98)	0.95 (0.91–0.98)	0.77 (0.65–0.90)
**Cancer**	62,231	1.06 (1.05–1.07)	1.05 (1.05–1.06)	1.29 (1.24–1.34)
**Bladder cancer**	633	1.08 (1.00–1.17)	1.07 (0.99–1.16)	1.39 (0.95–2.03)
**Brain cancer**	2,243	1.00 (0.95–1.04)	1.00 (0.96–1.04)	0.99 (0.81–1.22)
**Breast cancer**	11,429	1.00 (0.98–1.02)	1.00 (0.98–1.02)	1.00 (0.92–1.10)
**Breast cancer, <50 y.o.**	2,568	1.00 (0.96–1.04)	1.00 (0.96–1.04)	1.01 (0.84–1.21)
**Breast cancer, ≥50 y.o.**	8,861	1.00 (0.98–1.02)	1.00 (0.98–1.02)	1.00 (0.90–1.12)
**Colorectal cancer**	6,663	1.02 (1.00–1.05)	1.02 (0.99–1.04)	1.09 (0.97–1.23)
**Gallbladder cancer**	2,453	1.12 (1.08–1.17)	1.11 (1.06–1.15)	1.63 (1.35–1.96)
**Kidney cancer**	1,965	1.12 (1.08–1.17)	1.11 (1.07–1.16)	1.67 (1.36–2.06)
**Liver cancer**	1,359	1.10 (1.05–1.16)	1.09 (1.04–1.15)	1.52 (1.18–1.96)
**Lung cancer**	7,558	1.12 (1.09–1.14)	1.11 (1.09–1.13)	1.65 (1.48–1.83)
**Lymphatic cancer**	5,150	1.06 (1.03–1.09)	1.05 (1.02–1.08)	1.27 (1.11–1.45)
**Malignant melanoma**	1,103	1.04 (0.98–1.11)	1.04 (0.98–1.11)	1.23 (0.92–1.63)
**Oesophageal cancer**	429	1.07 (0.97–1.18)	1.07 (0.97–1.18)	1.40 (0.88–2.23)
**Ovarian cancer**	4,971	1.03 (1.01–1.06)	1.03 (1.00–1.06)	1.17 (1.02–1.34)
**Pancreatic cancer**	4,209	1.08 (1.04–1.11)	1.07 (1.04–1.11)	1.39 (1.20–1.61)
**Stomach cancer**	2,456	1.07 (1.03–1.11)	1.06 (1.01–1.10)	1.30 (1.07–1.57)
**Thyroid cancer**	272	1.08 (0.96–1.22)	1.07 (0.95–1.21)	1.39 (0.79–2.47)
**Uterine cancer**	3,521	1.10 (1.07–1.14)	1.09 (1.06–1.13)	1.52 (1.30–1.77)
**Cervical cancer**	1,938	1.07 (1.03–1.12)	1.05 (1.01–1.10)	1.29 (1.04–1.58)
**Endometrial cancer**	834	1.15 (1.08–1.23)	1.14 (1.07–1.22)	1.89 (1.38–2.59)

^a^HR from Cox regression of mothers’ mortality per SD (2.90 kg/m^2^) of sons’ BMI (*N* = 1,013,083). BMI was preadjusted for secular trends, conscription office, and age at examination. Each mother’s age was the time axis. This can be interpreted as the numerator of the ratio estimate for IV analyses.

^b^HR from Cox regression of mothers’ mortality per SD (2.90 kg/m^2^) of sons’ BMI (*N* = 1,013,083). BMI was preadjusted for secular trends, conscription office, age at examination, and both educational and occupational SEI. Each mother’s age was the time axis. This can be interpreted as the numerator of the ratio estimate for IV analyses.

^c^HR from Cox regression of mothers’ mortality per SD (2.90kg/m^2^) of mothers’ BMI, using a two-sample IV approach with sons’ BMI as the instrument for mothers’ BMI, adjusted for educational and occupational SEI, assuming that the mother–son association in BMI was equivalent to the father–son association in the subset of data with fathers’ BMI (*N* = 68,886).

**Abbreviations:** CHD, coronary heart disease; CI, confidence interval; CVD, cardiovascular disease; HR, hazard ratio; IV, instrumental variable; SD, standard deviation; SEI, socioeconomic index; y.o., years old

## Discussion

### Principal findings

Our analysis showed that higher BMI is likely to cause a greater risk of mortality from all-causes, CVDs, stroke, diabetes, respiratory diseases, overall cancer mortality, 9 site-specific cancers in men (bladder, colorectum, gallbladder, kidney, liver, lung, lymphatic system, pancreas, and stomach) and 11 site-specific cancers in women (gallbladder, kidney, liver, lung, lymphatic system, ovaries, pancreas, stomach, uterus, cervix, and endometrium). In mothers, there was evidence supporting the causal role of higher BMI on greater risk of mortality from kidney disease and lowered mortality from suicide. We also found evidence for positive associations between BMI and risk of mortality from respiratory disease and cancers of the prostate and lung, which has been inconsistently reported in the literature, suggesting that previous studies may suffer from confounding and, importantly, that the role of BMI in mortality from these causes may be underestimated.

### Comparison with other studies

Current results for all-cause mortality and mortality from CVDs, stroke, and diabetes are consistent with previous studies [4–7,31,32]. With each 5 kg/m^2^ higher BMI (i.e., transition between BMI categories), there was a 5% greater risk (95% CI: 4%–7%) of all-cause mortality in the largest meta-analysis including >30 million participants and approximately 3.7 million deaths [32] and, similarly, there was an approximately 40% greater risk of vascular mortality in >900,000 adults [4]. Scaling our current results for comparison, each 5 kg/m^2^ higher fathers’ BMI was associated with a 55% greater all-cause mortality risk (95% CI: 49%–59%), 94% great CVD mortality risk (HR 95% CI: 1.85–2.04) and more than a 2-fold greater CHD mortality risk (HR 95% CI: 2.01–2.27). Each 5 kg/m^2^ higher mothers’ BMI was associated with a 76% greater all-cause mortality risk (95% CI: 68%–83%) and more than a 2-fold greater risk of CVD (HR 95% CI: 2.13–2.47) and CHD (HR 95% CI: 2.60–3.22) mortality.

For cancer-specific mortality, many IV-derived effect estimates were in the same direction and of greater magnitude as those derived from previous large-scale meta-analyses and reviews focusing on both mortality outcomes and the development of specific cancers [4–8,31]. For example, higher BMI has been consistently associated with a greater risk of all-cause mortality and neoplastic mortality in multiple studies [4,5,32]. Our current results also provided some evidence for positive associations with the risk of mortality from respiratory disease, prostate cancer, and lung cancer, which have been inconsistently reported in the literature, suggesting that such observational results may be spurious, arising from confounding and, importantly, the role of BMI in mortality from these causes (and likely others) may be underestimated [4,6,33]. Indeed, the current literature is becoming more consistent with regards to the likely detrimental impact of higher BMI on the risk of aggressive prostate cancer [10]. Additionally, in the Million Women Study, higher BMI was associated with a greater risk of mortality from cancers of the endometrium, oesophagus, kidney, pancreas, lymphatic system, ovary, postmenopausal breast, and premenopausal colorectal cancer [6]. It is worth noting that cancers of the lymphatic system are very heterogeneous with regards to malignancy subtype definition and evidence supporting the likely impact of BMI on each subtype; therefore, larger studies that are able to separate cancer subtypes at scale are required to determine the specific impact of higher BMI on such outcomes [10]. Additionally, whilst it is likely that human papilloma virus (HPV) infection is the most potent risk factor for cervical cancer and the literature on BMI as a causal risk factor has been inconsistent, our study supports previous large-scale cohort studies and meta-analyses that have implicated a potential role of obesity in cervical cancer risk and mortality [6].

Our results are also consistent with the few studies using Mendelian randomization (MR; i.e., using genetic variation as IVs [34]) that have interrogated the causal impact of higher BMI on cancer-specific survival and mortality, which suggested that higher BMI reduced breast cancer–specific survival and increased prostate cancer mortality [13,35]. Furthermore, the causal relationship between higher BMI and lower mortality from suicide implied in the current analyses is consistent with a previous prospective study in the Nord-Trøndelag Health Study in Norway, which showed that suicide risk was lower with higher BMI (HR per SD higher BMI: 0.82; 95% CI: 0.68–0.98) [36]. However, there are inconsistencies in the literature [37]; therefore, these results should be taken with caution until replicated in prospective studies with causal methodologies and sample sizes comparable to the current analysis.

### Strengths

Our current analyses used comprehensive data from a large-scale, prospective Swedish cohort of over 1 million adults to assess the likely causal implications of higher BMI on mortality and extend our previous study of this relationship [20] in a variety of ways: we (i) used complementary one-sample (i.e., generating an IV estimate directly comparable to conventional Cox regression in the same data) and two-sample IV methodologies (i.e., increasing the precision of our causal estimates), rather than only the one-sample approach; (ii) presented HRs of mortality outcomes per unit higher parents’ BMI (as opposed to offspring BMI [20]) for better interpretation; and (iii) extended follow-up by 3 more years than previously reported, giving an additional 70,647 parent deaths. This greater sample size not only provided the statistical power to investigate several cancer subtypes and rarer causes of death with greater precision than has been done before (in the literature and by our previous efforts [20]) but allowed the investigation of a more comprehensive set of mortality outcomes, including endometrial, gallbladder, oesophageal, pancreatic, thyroid, and rectal cancers and malignant melanoma.

### Limitations

We and others have previously described the rationale for believing that using offspring exposure as an instrument for parents’ exposure is appropriate [20,23,27–29]. Whilst we were unable to test the association between sons’ and mothers’ BMI, most studies have found parents’ BMI to be strongly and similarly associated with sons’ BMI [38]. We confirmed this for fathers’ BMI here, implicating that sons’ BMI has good instrument strength. Generally, IV estimates may be biased if the behavioural, genetic, or socioeconomic factors confounding the observational association between parents’ BMI and parents’ mortality also influence sons’ BMI. This bias is stronger the weaker the instrument, so instrument strength of sons’ BMI limits the magnitude of this bias. Whilst most of the measured confounding factors were similarly associated with sons’ and parents’ BMI in this study, the associations between parents’ mortality and sons’ BMI were reassuringly negligibly influenced by adjustment for available confounders in conventional and IV analyses. Although we were unable to test whether the instrument was independent of unmeasured or residual confounding within this study, some of these confounding factors (e.g., fathers’ smoking) were more strongly associated with fathers’ BMI than with sons’ BMI. Therefore, the patterns of association observed between these confounding factors with parents’ and sons’ BMI were different, suggesting that the conventional and IV analyses are likely differentially biased by these factors. Thus, triangulation between these two analyses methods with differential biases, which showed similar estimates of association between BMI and mortality, provides more confidence in these current results [39]. Furthermore, the inherent correlation between sons’ and parental BMI measures and likely differences in intergenerational environment would be more likely to reduce bias in these analyses and, as such, is an improvement to observational measures alone.

We argue that these IV estimates are unlikely to be affected by reverse causality (an important source of bias), because a parent’s ill health is unlikely to directly affect their sons’ BMI [20,23]. It is also unlikely that sons’ BMI could directly affect parents' mortality [23]. Thus, whilst the IV methodology used here cannot be considered gold standard for assessing causality, results add to existing literature within this context.

More broadly, BMI is unable to distinguish fat from lean mass, a property of which has been suggested to explain why overweight individuals show the lowest risk of mortality [40]. Additionally, these results are applicable only to mortality and not to incidence or progression of the causes (i.e., cancer or CVD) of mortality analysed and many of the outcomes analyses are heterogeneous. Therefore, further large studies with accurate measures of body fatness (e.g., with dual-energy X-ray absorptiometry) and more refined measures of heterogeneous outcome diagnosis are required to disentangle the mechanisms by which higher BMI causes greater mortality risk and to provide more specific targets for population-level intervention. Lastly, given the sample size of complete data currently available (approximately 68,000 father–son pairs, representing fewer than 2,500 deaths), we were unable to analyse the nonlinear relationship between BMI and mortality, for which larger samples are required to fully understand the pattern of association between BMI and mortality over the full distribution of BMI.

## Conclusions

Higher BMI is likely to cause a greater risk of several common causes of death, including cancers of which the global incidence is increasing. We also found positive effects of BMI with mortality from respiratory disease, and cancers of the prostate and lung, which is inconsistently reported in the literature, suggesting that existing observational studies may suffer from confounding and, importantly, the role of BMI in mortality from these specific causes may be underestimated. These findings therefore further support efforts for reducing the rising population trends of obesity.

## Supporting information

S1 STROBE Checklist Checklist of items that should be included in reports of observational studies. STROBE, Strengthening the reporting of Observational studies in epidemiology.
(DOC)

S1 Text Supporting methods. (DOCX)

S1 Table ICD codes used to define binary outcomes. ICD, international classification of diseases.
(DOCX)

S2 Table Characteristics of the sons and parents according to quintiles of fathers’ BMI (in the subset with fathers’ BMI data). BMI, body mass index.
(DOCX)

S3 Table Characteristics of the sons and parents according to quintiles of sons’ BMI (in the subset with fathers’ BMI data). BMI, body mass index.
(DOCX)

S4 Table A comparison of models for estimating associations of fathers’ mortality per SD (2.90 kg/m^2^) of BMI at conscription (sons’ or own) (*N* = 68,886). BMI, body mass index, SD, standard deviation.
(DOCX)

S5 Table Comparison of HR for mothers’ and fathers’ mortality per SD of sons’ BMI (restricting to the sons contributing to analyses of both parents). BMI, body mass index; HR, hazard ratio; SD, standard deviation.
(DOCX)

## Abbreviations

BMI: body mass index
CHD: coronary heart disease
CI: confidence interval
CVD: cardiovascular disease
HPV: human papilloma virus
HR: hazard ratio
ICD: international classification of diseases
ID: identity number
IV: instrumental variable
MR: Mendelian randomization
NHS: National Health Service
NIHR: National Institute for Health Research
SD: standard deviation
SEI: socioeconomic index
