# Supplementary material for: Variation of all-cause and cause-specific mortality with body mass index in one million Swedish parent-son pairs: An instrumental variable analysis
Source: PLoS Med. 2019 Aug 9;16(8):e1002868. doi: 10.1371/journal.pmed.1002868 (PMC6688790; doi:10.1371/journal.pmed.1002868)
Supplement: S1 STROBE Checklist — STROBE, Strengthening the reporting of Observational studies in epidemiology. (DOC) [file pmed.1002868.s001.doc]

STROBE Statement—checklist of items that should be included in reports of observational studies

|  | Item No | Recommendation | Section and paragraph number |
| --- | --- | --- | --- |
| **Title and abstract** | 1 | (*a*) Indicate the study’s design with a commonly used term in the title or the abstract | Title.  Abstract: Background, and Methods/Findings sections |
|  |  | (*b*) Provide in the abstract an informative and balanced summary of what was done and what was found | Abstract: Background, Methods/Findings and Conclusions sections |
| Introduction | | |  |
| Background/rationale | 2 | Explain the scientific background and rationale for the investigation being reported | Paragraphs 1-3 |
| Objectives | 3 | State specific objectives, including any prespecified hypotheses | Paragraph 3 |
| Methods | | |  |
| Study design | 4 | Present key elements of study design early in the paper | Paragraphs 3-10 |
| Setting | 5 | Describe the setting, locations, and relevant dates, including periods of recruitment, exposure, follow-up, and data collection | Paragraph 4 |
| Participants | 6 | (*a*) *Cohort study*—Give the eligibility criteria, and the sources and methods of selection of participants. Describe methods of follow-up  *Case-control study*—Give the eligibility criteria, and the sources and methods of case ascertainment and control selection. Give the rationale for the choice of cases and controls  *Cross-sectional study*—Give the eligibility criteria, and the sources and methods of selection of participants | (a) Paragraph 4, Fig 1 and S1 Text (paragraphs 1-2). |
|  |  | (*b*) *Cohort study*—For matched studies, give matching criteria and number of exposed and unexposed  *Case-control study*—For matched studies, give matching criteria and the number of controls per case | (b) Paragraph 4, Fig 1 and S1 Text (paragraphs 1-2) and S1 Table. |
| Variables | 7 | Clearly define all outcomes, exposures, predictors, potential confounders, and effect modifiers. Give diagnostic criteria, if applicable | Paragraphs 5-6 and S1 Text (paragraphs 3-4). |
| Data sources/ measurement | 8* | For each variable of interest, give sources of data and details of methods of assessment (measurement). Describe comparability of assessment methods if there is more than one group | Paragraphs 5-6 and S1 Text (paragraphs 3-4). |
| Bias | 9 | Describe any efforts to address potential sources of bias | Paragraphs 6-9 and S1 Text (5-7). |
| Study size | 10 | Explain how the study size was arrived at | Paragraphs 4-6 and S1 Text (paragraphs 1-4). |
| Quantitative variables | 11 | Explain how quantitative variables were handled in the analyses. If applicable, describe which groupings were chosen and why | Paragraph 5-6 and S1 Text (paragraphs 1-6). |
| Statistical methods | 12 | (*a*) Describe all statistical methods, including those used to control for confounding | Paragraphs 5-9 and S1 Text (paragraphs 5-7). |
|  |  | (*b*) Describe any methods used to examine subgroups and interactions | n/a |
|  |  | (*c*) Explain how missing data were addressed | Paragraphs 5-9 and S1 Text (paragraphs 5-7). |
|  |  | (*d*) *Cohort study*—If applicable, explain how loss to follow-up was addressed  *Case-control study*—If applicable, explain how matching of cases and controls was addressed  *Cross-sectional study*—If applicable, describe analytical methods taking account of sampling strategy | n/a |
|  |  | (*e*) Describe any sensitivity analyses | n/a |

Continued on next page

| Results | | | Section and paragraph number |
| --- | --- | --- | --- |
| Participants | 13* | (a) Report numbers of individuals at each stage of study—eg numbers potentially eligible, examined for eligibility, confirmed eligible, included in the study, completing follow-up, and analysed | Paragraph 4, Fig 1 and S1 Text (paragraphs 1-2) and Paragraph 11. |
|  |  | (b) Give reasons for non-participation at each stage | Paragraph 4, Fig 1 and S1 Text (paragraphs 1-2). |
|  |  | (c) Consider use of a flow diagram | Paragraph 4, Fig 1 and S1 Text (paragraphs 1-2). |
| Descriptive data | 14* | (a) Give characteristics of study participants (eg demographic, clinical, social) and information on exposures and potential confounders | Paragraph 11 and Table 1; S1 Table -S3 Table. |
|  |  | (b) Indicate number of participants with missing data for each variable of interest | Fig 1, Table 1. |
|  |  | (c) *Cohort study*—Summarise follow-up time (eg, average and total amount) | Paragraph 4, Fig 1 and S1 Text (paragraphs 1-2). |
| Outcome data | 15* | *Cohort study*—Report numbers of outcome events or summary measures over time | S1 Table. |
|  |  | *Case-control study—*Report numbers in each exposure category, or summary measures of exposure | n/a |
|  |  | *Cross-sectional study—*Report numbers of outcome events or summary measures | n/a |
| Main results | 16 | (*a*) Give unadjusted estimates and, if applicable, confounder-adjusted estimates and their precision (eg, 95% confidence interval). Make clear which confounders were adjusted for and why they were included | Paragraph 12-13 |
|  |  | (*b*) Report category boundaries when continuous variables were categorized | Table 1 and S1 Text (paragraphs 3-4). |
|  |  | (*c*) If relevant, consider translating estimates of relative risk into absolute risk for a meaningful time period | n/a |
| Other analyses | 17 | Report other analyses done—eg analyses of subgroups and interactions, and sensitivity analyses | n/a |
| Discussion | | |  |
| Key results | 18 | Summarise key results with reference to study objectives | Paragraph 16-20 |
| Limitations | 19 | Discuss limitations of the study, taking into account sources of potential bias or imprecision. Discuss both direction and magnitude of any potential bias | Paragraphs 21-23 |
| Interpretation | 20 | Give a cautious overall interpretation of results considering objectives, limitations, multiplicity of analyses, results from similar studies, and other relevant evidence | Paragraphs 16-23 |
| Generalisability | 21 | Discuss the generalisability (external validity) of the study results | Paragraphs 21-23 |
| Other information | | |  |
| Funding | 22 | Give the source of funding and the role of the funders for the present study and, if applicable, for the original study on which the present article is based | Paragraph 26 |

*Give information separately for cases and controls in case-control studies and, if applicable, for exposed and unexposed groups in cohort and cross-sectional studies.

**Note:** An Explanation and Elaboration article discusses each checklist item and gives methodological background and published examples of transparent reporting. The STROBE checklist is best used in conjunction with this article (freely available on the Web sites of PLoS Medicine at http://www.plosmedicine.org/, Annals of Internal Medicine at http://www.annals.org/, and Epidemiology at http://www.epidem.com/). Information on the STROBE Initiative is available at www.strobe-statement.org.
