# Supplementary material for: Variation of all-cause and cause-specific mortality with body mass index in one million Swedish parent-son pairs: An instrumental variable analysis
Source: PLoS Med. 2019 Aug 9;16(8):e1002868. doi: 10.1371/journal.pmed.1002868 (PMC6688790; doi:10.1371/journal.pmed.1002868)
Supplement: S1 Text — (DOCX) [file pmed.1002868.s002.docx]

**S1 Text**

**Supporting Methods**

*Study genesis and analysis plan*

Before the study began, the informal analysis plan was to re-address the research question addressed in our previous study (1) using extended follow-up and several more powerful instrumental variable (IV) methods subsequently applied in our other studies using offspring exposures as instrumental variables for those variables in their parents (2,3). The extension to follow-up exceeded three years, providing more than 70,000 additional deaths and the ability to investigate a more comprehensive set of mortality outcomes, including rarer causes of death and cancer subtypes, than was previously analysed. The main *a priori* extensions to the methods, compared to the previous study of this dataset, were (i) use of ratio-method IV to estimate IV hazard ratios (in addition to two-stage IV estimating IV rate ratios), (ii) use of two-sample IV, making maximum use of the data available (including maternal mortality) and considerably increasing power (iii) formal comparison of analogous (one-sample) IV and conventional estimates. During the analysis, we decided to use Durbin-Wu Hausman (DWH) tests rather than the originally intended bootstraps to compare analogous IV and conventional estimates. This was for their improved speed and repeatability.

*Study population and data linkage*

The Swedish Multi-Generation Register was used to identify all 1,629,396 boys born in Sweden between 1951 and 1980, and their biological parents, using the unique individual identity numbers given to all citizens and individuals with permanent permission to live in Sweden(1,2). Sons’ BMI was calculated by dividing weight (kg) by the square of height (m^2^). Conscription data were completely missing for around 15% of sons, mainly because of accidental loss of data from 1978, 1984 and 1985 owing to changes in data management at the conscription authority (**Figure 1**, main text).

The parents’ identity numbers were matched to the Swedish Cause of Death Register, providing the dates and causes of parents’ deaths between 1/1/1961-31/12/2004. Emigration records were available, and we assumed that parents who were not dead and had not emigrated by 31 December 2004 were still alive at this time. The mothers of 231,423 sons and the fathers of 443,091 sons had been recorded dead by that date. The conscription examination records included data for 89,311 father-son pairs, with fathers having undergone conscription examinations for height and weight between 1/9/1969 and 1/5/1991. Data on smoking habits were available for 23,018 fathers undergoing conscription examinations between 1969 and 1970 and for 36,273 sons. Father’s BMI was calculated in the same way as son’s BMI. Data on mothers’ height, weight and smoking were not available for this study.

Data on smoking habits were available for 23,018 fathers undergoing conscription examinations between 1969 and 1970 and for 36,273 sons. Father’s BMI was calculated in the same was as with son’s BMI. Data on mothers’ height, weight and smoking were not available for this study. The Swedish Population and Housing Census provided data on parents’ educational level and occupational socioeconomic index (SEI) in 1970 and 1990. We took the higher of the 1970 and 1990 values for educational level and classified it into five levels: <9 years; 9–10 years; full secondary; higher; and missing (7% of fathers and 3% of mothers). A binary variable derived from this distinguished those with and without at least ten years’ education (with the final category omitted as missing data). We also classified parents according to five mutually exclusive categories of occupational SEI: high/intermediate non-manual; low non-manual; skilled manual; unskilled manual; and other/missing (19% of fathers and 30% of mothers). We used the 1970 value for parents born before 1935 and the 1990 value for parents born later. A binary variable derived from this distinguished those in manual and non-manual employment (with the final category omitted as missing data).

Sons were removed from the analysis if they lacked information on BMI, date of birth, conscription office or examination date. They were not used in the analysis of parents’ mortality if their parents lacked plausible information on date of birth or date of death, or if their parents were never at risk during the follow-up period. For each parent, the follow-up period ran from the latter of their son’s birth and 1/1/1961 until the parent’s death, parent’s emigration or 31/12/2004. To avoid violation of independence assumptions, data were restricted to one randomly chosen son per parent and, where possible, the same son was chosen for both parents. This gave a sample of 996,898 father–son pairs and 1,013,083 mother–son pairs (for 1,036,817 sons and including 973,164 complete trios) (Figure 1, main text).

*Statistical analysis*

BMI values for fathers and sons were adjusted for age, conscription office and secular trends before all analyses (BMI was not available for mothers). This was done by taking residuals from linear regression of the fathers’ or sons’ BMI (separately) against their age at examination (cubic spline with 7 knots at percentiles 2.5, 18.3, 34.2, 50, 65.8, 81.7, 97.5)(4), date of birth (cubic spline with 7 knots at percentiles 2.5, 18.3, 34.2, 50, 65.8, 81.7, 97.5) and conscription office (six-level categorical variable). These residuals were further divided by the residual standard deviation (SD; 2.90 kg/m^2^), using the residual SD from the sons to keep the units the same for fathers and sons.

As a comparison to the main instrumental variable analysis, alternative IV estimates of the HR per SD of fathers’ BMI were made using Stata’s qvf command to conduct Poisson regression within strata of fathers’ ages (category thresholds at 30, 40, 45 and 50 years) with time at risk as an offset (equivalent to a stratified exponential parametric survival model, with constant hazard within each stratum of age) and sons’ BMI as the instrument (presented here, in the Supporting Information).

We generated alternative IV estimates using Stata’s qvf command to conduct Poisson regression within strata of fathers’ ages (category thresholds at 30, 40, 45 and 50 years) with time-at-risk as an offset (equivalent to a stratified exponential parametric survival model, with constant hazard within each stratum of age) and sons’ BMI as the instrument (presented in Supporting Information).

**References**

1. Davey Smith G, Sterne JA, Fraser A, Tynelius P, Lawlor DA, Rasmussen F. The association between BMI and mortality using offspring BMI as an indicator of own BMI: large intergenerational mortality study. BMJ. 2009;339;b5043; doi: 10.1136/bmj.b5043.

2. Carslake D, Fraser A, Davey Smith G, May M, Palmer T, Sterne J, et al. Associations of mortality with own height using son's height as an instrumental variable. Econ Hum Biol. 2013;11(3):351-9. doi: http://dx.doi.org/10.1016/j.ehb.2012.04.003.

3. Carslake D, Davey Smith G, Gunnell D, Davies N, Nilsen TIL, Romundstad P. Confounding by ill health in the observed association between BMI and mortality: evidence from the HUNT Study using offspring BMI as an instrument. Int J Epidemiol. 2017;47(3):760-70. doi: 10.1093/ije/dyx246.

4. Harrell FE. Regression modeling stategies with applications to linear models, logistic regression and survival analysis. New York: Springer, 2001.
