# Supplementary material for: Variation of all-cause and cause-specific mortality with body mass index in one million Swedish parent-son pairs: An instrumental variable analysis
Source: PLoS Med. 2019 Aug 9;16(8):e1002868. doi: 10.1371/journal.pmed.1002868 (PMC6688790; doi:10.1371/journal.pmed.1002868)
Supplement: S1 Table — ICD, international classification of diseases. (DOCX) [file pmed.1002868.s003.docx]

**S1 Table: ICD codes used to define binary outcomes.**

| Diagnosis | ICD 7 | ICD 8 | ICD 9 | ICD 10 |
| --- | --- | --- | --- | --- |
| Cardiovascular disease | 330-33499 400-41699 420-42299 430-44799 450-46899 782-78299 | 390-40499 410-41499 420-42909 4299-43899 440-45899 782-78299 | 390-40599 410-43899 440-45999 | G45-G4599 I00-I1599 I20-I5299 I60-I9999 |
| Coronary heart disease | 420-42099 422-42299 450-45099 | 410-41499 4299-42999 | 410-41499 4292-42929 | I20-I2599 I516-I5169 |
| Aortic aneurysm | 451-45199 | 441-44199 | 441-44199 | I71-I7199 |
| Stroke | 306-30699 330-33499 352-35299 | 2930-29319 344-34499 430-43899 | 2904-29049 342-34299 344-34499 430-43899 | F01-F0199 G45-G4599 G81-G8199 G83-G8399 I60-I6999 |
| Diabetes | 260-26099 | 250-25099 | 250-25099 | E10-E1499 |
| Kidney disease | 590-60499 | 580-59499 | 580-59499 | N00-N2999 |
| Respiratory diseases | 240-24199 470-52799 | 460-51999 | 460-51999 | J00-J9999 |
| External causes | E800-E9999 | E800-E9999 | E800-E9999 | V01-Y9899 |
| Suicide | E970-E9799 | E950-E9599 | E950-E9599 | X60-X8499 |
| Cancer | 140-20799 | 140-20799 | 140-20939 | C00-C9799 |
| Bladder cancer | 1810-18109 | 188-18899 | 188-18899 | C67-C6799 |
| Brain cancer | 193-19399 | 191-19299 | 191-19299 | C71-C7299 |
| Breast cancer | 170-17099 | 174-17499 | 174-17599 | C50-C5099 |
| Colorectal cancer | 153-15499 | 153-15499 | 153-15499 | C18-C2199 |
| Gallbladder cancer | 1551-15519 | 156-15699 | 156-15699 | C23-C2499 |
| Kidney cancer | 180-18099 | 1890-18929 | 1890-18929 | C64-C6699 |
| Liver cancer | 155-15509 1552-15599 | 155-15599 | 155-15599 | C22-C2299 |
| Lung cancer | 162-16219 1628-16399 | 162-16299 | 162-16299 | C33-C3499 |
| Lymphatic cancer | 200-20799 | 200-20799 | 200-20899 | C81-C9699 |
| Malignant melanoma | 190-19099 | 172-17299 | 172-17299 | C43-C4399 |
| Oesophageal cancer | 150-15099 | 150-15099 | 150-15099 | C15-C1599 |
| Ovarian cancer | 175-17599 | 183-18399 | 183-18399 | C56-C5699 C570-C5749 |
| Prostate cancer | 177-17799 | 185-18599 | 185-18599 | C61-C6199 |
| Pancreatic cancer | 157-15799 | 157-15799 | 157-15799 | C25-C2599 |
| Stomach cancer | 151-15199 | 151-15199 | 151-15199 | C16-C1699 |
| Testicular cancer | 178-17899 | 186-18699 | 186-18699 | C62-C6299 |
| Thyroid cancer | 194-19499 | 193-19399 | 193-19399 | C73-C7399 |
| Uterine cancer | 171-17499 | 180-18299 | 179-18289 | C53-C5599 |
| Cervical cancer | 171-17199 | 180-18099 | 180-18099 | C53-C5399 |
| Endometrial cancer | 172-17299 | 1820-18209 | 1820-18209 | C54-C5499 |

*ICD, International Classification of Disease*

*ICD codes were recorded in 3-5 characters. Ranges are alphabetical, not numerical. For example, 12288<12289<1229<123<1230<12300<12301 and the range “123-12399” includes any 3-5 digit codes starting with “123”.*
