# Supplementary material for: Variation of all-cause and cause-specific mortality with body mass index in one million Swedish parent-son pairs: An instrumental variable analysis
Source: PLoS Med. 2019 Aug 9;16(8):e1002868. doi: 10.1371/journal.pmed.1002868 (PMC6688790; doi:10.1371/journal.pmed.1002868)
Supplement: S2 Table — BMI, body mass index. (DOCX) [file pmed.1002868.s004.docx]

**S2 Table: Characteristics of the sons and parents according to quintiles of fathers’ BMI (in the subset with fathers’ BMI data).**

|  |  | Quintile of fathers’ BMI | | | | | Mean difference or odds ratio (95% CI) | N |
| --- | --- | --- | --- | --- | --- | --- | --- | --- |
| Subject | Variable | 1^st^ | 2^nd^ | 3^rd^ | 4^th^ | 5^th^ |  |  |
| Sons | Unadjusted BMI^a,b^ (kg/m^2^) | 21.2 | 21.9 | 22.3 | 22.9 | 23.8 | 1.09 (1.07, 1.12) | 68,886 |
|  | Height^a,b^ (cm) | 179.5 | 179.5 | 179.4 | 179.4 | 179.4 | -0.09 (-0.14, -0.03) | 68,886 |
|  | Date of birth^a^ | 1977.8 | 1977.8 | 1977.8 | 1977.8 | 1977.7 | -0.03 (-0.05, -0.01) | 68,886 |
| Fathers | Unadjusted BMI^a,b^ (kg/m^2^) | 18.3 | 19.9 | 20.9 | 22.2 | 24.9 | 2.90 (2.90, 2.90) | 68,886 |
|  | Height^a,b^ (cm) | 178.6 | 178.3 | 178.2 | 178.1 | 178.0 | -0.23 (-0.29, -0.18) | 68,886 |
|  | Smokers^b,c^ (%) | 71% | 67% | 64% | 61% | 61% | 0.85 (0.82, 0.89) | 13,860 |
|  | Age at sons’ birth (years)^a^ | 24.0 | 24.2 | 24.2 | 24.1 | 24.0 | -0.03 (-0.06, -0.01) | 68,886 |
|  | Educated > 10 years^c^ (%) | 65% | 67% | 67% | 67% | 63% | 0.93 (0.92, 0.95) | 67,301 |
|  | In non-manual work^c^ (%) | 43% | 45% | 44% | 43% | 39% | 0.92 (0.90, 0.93) | 59,051 |
| Mothers | Date of birth | 1955.1 | 1954.9 | 1954.7 | 1954.7 | 1954.6 | -0.17 (-0.20, -0.14) | 68,886 |
|  | Age at sons’ birth (years)^a^ | 22.7 | 22.9 | 23.1 | 23.1 | 23.1 | 0.14 (0.11, 0.17) | 68,886 |
|  | Educated > 10 years^c^ (%) | 71% | 72% | 73% | 72% | 69% | 0.95 (0.93, 0.97) | 67,530 |
|  | In non-manual work^c^ (%) | 43% | 45% | 46% | 45% | 41% | 0.94 (0.92, 0.96) | 57,183 |

*BMI, body mass index; CI, confidence interval; SD, standard deviation*

*^a^Continuous variables are summarised as means in each quintile and linear regression produced mean differences per SD (2.90 kg/m^2^) of BMI pre-adjusted for each father’s age at examination, conscription office and secular trends (date of birth).*

*^b^Measured at pre-conscription medical examination. Smoking was only recorded at examinations in 1969-1970.*

*^c^Binary variables are summarised as percentages in each quintile and logistic regression produced odds ratios per SD (2.90 kg/m^2^) of BMI pre-adjusted for each father’s age at examination, conscription office and secular trends (date of birth).*
