# Supplementary material for: Variation of all-cause and cause-specific mortality with body mass index in one million Swedish parent-son pairs: An instrumental variable analysis
Source: PLoS Med. 2019 Aug 9;16(8):e1002868. doi: 10.1371/journal.pmed.1002868 (PMC6688790; doi:10.1371/journal.pmed.1002868)
Supplement: S3 Table — BMI, body mass index. (DOCX) [file pmed.1002868.s005.docx]

**S3 Table: Characteristics of the sons and parents according to quintiles of sons’ BMI (in the subset with fathers’ BMI data).**

|  |  | Quintile of sons’ BMI | | | | | Mean difference or odds ratio (95% CI) |  |
| --- | --- | --- | --- | --- | --- | --- | --- | --- |
| Subject | Variable | 1^st^ | 2^nd^ | 3^rd^ | 4^th^ | 5^th^ |  | N |
| Sons | Unadjusted BMI^a,b^ (kg/m^2^) | 19.0 | 20.8 | 21.9 | 23.3 | 27.1 | 2.90 (2.90, 2.90) | 68,886 |
|  | Height^a,b^ (cm) | 179.8 | 179.5 | 179.4 | 179.2 | 179.3 | -0.12 (-0.16, -0.07) | 68,886 |
| Fathers | Unadjusted BMI^a,b^ (kg/m^2^) | 20.3 | 20.9 | 21.2 | 21.6 | 22.2 | 0.62 (0.60, 0.63) | 68,886 |
|  | Height^a,b^ (cm) | 178.4 | 178.3 | 178.2 | 178.1 | 178.2 | -0.09 (-0.13, -0.05) | 68,886 |
|  | Date of birth^a^ | 1953.7 | 1953.6 | 1953.6 | 1953.6 | 1953.7 | 0.04 (0.03, 0.05) | 68,886 |
|  | Smokers^b,c^ (%) | 62% | 65% | 63% | 66% | 68% | 1.08 (1.05, 1.12) | 13,860 |
|  | Age at sons’ birth (years)^a^ | 24.3 | 24.1 | 24.1 | 24.0 | 24.0 | -0.06 (-0.08, -0.05) | 68,886 |
|  | Educated > 10 years^c^ (%) | 67% | 67% | 66% | 66% | 63% | 0.94 (0.92, 0.95) | 67,301 |
|  | In non-manual work^c^ (%) | 44% | 45% | 44% | 43% | 39% | 0.91 (0.90, 0.92) | 59,051 |
| Mothers | Date of birth | 1954.8 | 1954.8 | 1954.7 | 1954.7 | 1954.9 | 0.06 (0.04, 0.08) | 68,886 |
|  | Age at sons’ birth (years)^a^ | 23.1 | 23.0 | 23.0 | 22.9 | 22.8 | -0.08 (-0.11, -0.06) | 68,886 |
|  | Educated > 10 years^c^ (%) | 72% | 73% | 73% | 72% | 69% | 0.93 (0.92, 0.95) | 67,530 |
|  | In non-manual work^c^ (%) | 44% | 45% | 46% | 44% | 40% | 0.93 (0.92, 0.94) | 57,183 |

*BMI, body mass index; CI, confidence interval; SD, standard deviation*

*^a^Continuous variables are summarised as means in each quintile and linear regression produced mean differences per SD (2.90 kg/m^2^) of BMI pre-adjusted for each son’s age at examination, conscription office and secular trends (date of birth).*

*^b^Measured at pre-conscription medical examination. Smoking was only recorded at examinations in 1969-1970.*

*^c^Binary variables are summarised as percentages in each quintile and logistic regression produced odds ratios per SD (2.90 kg/m^2^) of BMI pre-adjusted for each son’s age at examination, conscription office and secular trends (date of birth).*
