# Supplementary material for: Variation of all-cause and cause-specific mortality with body mass index in one million Swedish parent-son pairs: An instrumental variable analysis
Source: PLoS Med. 2019 Aug 9;16(8):e1002868. doi: 10.1371/journal.pmed.1002868 (PMC6688790; doi:10.1371/journal.pmed.1002868)
Supplement: S4 Table — BMI, body mass index, SD, standard deviation. (DOCX) [file pmed.1002868.s006.docx]

**S4 Table: A comparison of models for estimating associations of fathers’ mortality per SD (2.90 kg/m^2^) of BMI at conscription (sons’ or own) (N=68,886)**

| Cause of death | Deaths | IV HR^a^ (95% CI) per SD of own BMI, adjusted |
| --- | --- | --- |
| All cause | 2,436 | 1.10 (0.93, 1.30) |
| Cardiovascular disease | 436 | 1.53 (1.06, 2.20) |
| Coronary heart disease | 243 | 1.90 (1.19, 3.02) |
| Stroke | 89 | 1.55 (0.68, 3.51) |
| Respiratory diseases | 42 | 0.50 (0.21, 1.16) |
| External causes | 1,109 | 0.92 (0.71, 1.19) |
| Suicide | 495 | 0.69 (0.45, 1.05) |
| Cancer | 444 | 1.22 (0.84, 1.79) |
| Brain cancer | 66 | 0.69 (0.21, 2.25) |
| Colorectal cancer | 43 | 1.56 (0.64, 3.78) |
| Lung cancer | 63 | 0.97 (0.36, 2.60) |
| Lymphatic cancer | 67 | 1.12 (0.42, 2.97) |

*BMI, body mass index; CI, confidence interval; HR, hazard ratio); IV, instrumental variable; SD, standard deviation; SEI, socioeconomic index.*

*^a^HR from a two-stage IV estimate using sons’ BMI as the instrument for fathers’ BMI and a father’s age-stratified Poisson regression with time at risk as an offset in Stata’s qvf command.*

*These analyses were all restricted entirely to those fathers with BMI data (N=68,886) and to conditions causing at least 40 fathers’ deaths within this subset. BMI was pre-adjusted for secular trends, conscription office, age at examination and both educational and occupational SEI*
