# Supplementary material for: Variation of all-cause and cause-specific mortality with body mass index in one million Swedish parent-son pairs: An instrumental variable analysis
Source: PLoS Med. 2019 Aug 9;16(8):e1002868. doi: 10.1371/journal.pmed.1002868 (PMC6688790; doi:10.1371/journal.pmed.1002868)
Supplement: S5 Table — BMI, body mass index; HR, hazard ratio; SD, standard deviation. (DOCX) [file pmed.1002868.s007.docx]

**S5 Table: Comparison of hazard ratios for mothers’ and fathers’ mortality per SD of sons’ BMI (restricting to the sons contributing to analyses of both parents).**

| Cause of death | Fathers’ deaths | Mothers’ deaths | HR^a^ (95% CI) for fathers’ mortality per SD of sons’ BMI | HR^a^ (95% CI) for mothers’ mortality per SD of sons’ BMI | P-value for comparison of HR^b^ |
| --- | --- | --- | --- | --- | --- |
| All cause | 274,910 | 145,487 | 1.05 (1.05, 1.06) | 1.07 (1.07, 1.08) | <0.001 |
| Cardiovascular disease | 124,539 | 48,529 | 1.08 (1.08, 1.09) | 1.11 (1.10, 1.12) | <0.001 |
| Coronary heart disease | 79,988 | 22,895 | 1.09 (1.09, 1.10) | 1.14 (1.12, 1.15) | <0.001 |
| Aortic aneurysm | 4,900 | 1,454 | 1.08 (1.05, 1.11) | 1.10 (1.04, 1.16) | 0.573 |
| Stroke | 21,527 | 13,840 | 1.04 (1.03, 1.06) | 1.05 (1.03, 1.06) | 0.764 |
| Diabetes | 4,149 | 2,496 | 1.20 (1.17, 1.24) | 1.35 (1.30, 1.39) | <0.001 |
| Kidney disease | 1,943 | 1,091 | 1.04 (0.99, 1.09) | 1.18 (1.12, 1.25) | <0.001 |
| Respiratory diseases | 13,641 | 7,375 | 1.03 (1.01, 1.05) | 1.09 (1.06, 1.11) | <0.001 |
| External causes | 24,380 | 8,745 | 1.00 (0.99, 1.01) | 0.98 (0.96, 1.00) | 0.077 |
| Suicide | 9,086 | 3,540 | 0.98 (0.96, 1.00) | 0.95 (0.92, 0.98) | 0.122 |
| Cancer | 77,592 | 59,430 | 1.04 (1.03, 1.05) | 1.06 (1.05, 1.06) | 0.006 |
| Bladder cancer | 2,435 | 600 | 1.04 (1.00, 1.09) | 1.04 (0.96, 1.13) | 0.972 |
| Brain cancer | 3,037 | 2,144 | 1.01 (0.97, 1.05) | 1.00 (0.96, 1.04) | 0.739 |
| Colorectal cancer | 8,744 | 6,409 | 1.05 (1.03, 1.07) | 1.02 (0.99, 1.04) | 0.068 |
| Gallbladder cancer | 1,340 | 2,370 | 1.06 (1.01, 1.12) | 1.11 (1.07, 1.16) | 0.172 |
| Kidney cancer | 3,414 | 1,889 | 1.07 (1.03, 1.11) | 1.12 (1.07, 1.17) | 0.111 |
| Liver cancer | 2,145 | 1,301 | 1.13 (1.09, 1.18) | 1.09 (1.03, 1.15) | 0.265 |
| Lung cancer | 14,199 | 7,079 | 1.03 (1.01, 1.05) | 1.12 (1.10, 1.15) | <0.001 |
| Lymphatic cancer | 8,039 | 4,954 | 1.04 (1.02, 1.06) | 1.05 (1.02, 1.08) | 0.648 |
| Malignant melanoma | 1,826 | 1,054 | 1.00 (0.95, 1.05) | 1.04 (0.98, 1.11) | 0.270 |
| Oesophageal cancer | 1,686 | 404 | 1.03 (0.98, 1.08) | 1.06 (0.96, 1.18) | 0.537 |
| Pancreatic cancer | 5,144 | 4,031 | 1.08 (1.05, 1.11) | 1.07 (1.04, 1.10) | 0.760 |
| Stomach cancer | 4,825 | 2,354 | 1.06 (1.03, 1.09) | 1.05 (1.01, 1.10) | 0.771 |
| Thyroid cancer | 221 | 260 | 0.99 (0.86, 1.14) | 1.07 (0.94, 1.21) | 0.475 |

*BMI, body mass index; CI, confidence interval; HR, hazard ratio; SD, standard deviation; SEI, socioeconomic index.*

*^a^From Cox regressions of mothers’ or fathers’ mortality per SD (2.90 kg/m^2^) of sons’ BMI, adjusted for educational and occupational SEI. Sons’ BMI was adjusted before analysis for secular trends, conscription office and age at examination. Analyses were restricted to the 973,164 mother-father-son trios, where the same sons were used to analyse mothers and fathers.*

*^b^P values comparing HR for mothers’ and fathers’ mortality were estimated from 1000 bootstrap resamples.*
